# Interplay between Fungal Infection and Bacterial Associates in the Wax Moth *Galleria mellonella* under Different Temperature Conditions

**DOI:** 10.3390/jof6030170

**Published:** 2020-09-10

**Authors:** Vadim Yu Kryukov, Elena Kosman, Oksana Tomilova, Olga Polenogova, Ulyana Rotskaya, Maksim Tyurin, Tatyana Alikina, Olga Yaroslavtseva, Marsel Kabilov, Viktor Glupov

**Affiliations:** 1 Institute of Systematics and Ecology of Animals SB RAS, Frunze str. 11, 630091 Novosibirsk, Russia; vereshchagina86@gmail.com (E.K.); toksina@mail.ru (O.T.); ovp0408@yandex.ru (O.P.); ulyanar@mail.ru (U.R.); maktolt@mail.ru (M.T.); yarosl@inbox.ru (O.Y.); skif@eco.nsc.ru (V.G.); 2 Institute of Chemical Biology and Fundamental Medicine SB RAS, Lavrentiev av. 8, 630090 Novosibirsk, Russia; alikina@niboch.nsc.ru (T.A.); kabilov@niboch.nsc.ru (M.K.)

**Keywords:** insects, mycoses, spontaneous bacterioses, fungal–bacteria interactions, *Cordyceps militaris*, antimicrobial peptides

## Abstract

Various insect bacterial associates are involved in pathogeneses caused by entomopathogenic fungi. The outcome of infection (fungal growth or decomposition) may depend on environmental factors such as temperature. The aim of this study was to analyze the bacterial communities and immune response of *Galleria mellonella* larvae injected with *Cordyceps militaris* and incubated at 15 °C and 25 °C. We examined changes in the bacterial CFUs, bacterial communities (Illumina MiSeq 16S rRNA gene sequencing) and expression of immune, apoptosis, ROS and stress-related genes (qPCR) in larval tissues in response to fungal infection at the mentioned temperatures. Increased survival of larvae after *C. militaris* injection was observed at 25 °C, although more frequent episodes of spontaneous bacteriosis were observed at this temperature compared to 15 °C. We revealed an increase in the abundance of enterococci and enterobacteria in the midgut and hemolymph in response to infection at 25 °C, which was not observed at 15 °C. Antifungal peptide genes showed the highest expression at 25 °C, while antibacterial peptides and inhibitor of apoptosis genes were strongly expressed at 15 °C. Cultivable bacteria significantly suppressed the growth of *C. militaris*. We suggest that fungi such as *C. militaris* may need low temperatures to avoid competition with host bacterial associates.

## 1. Introduction

The development of infectious diseases in animals is often accompanied by the proliferation of complex concomitant microorganisms in addition to the development of the main pathogen. In particular, mycoses of insects may develop as mixed infections when opportunistic bacteria are actively involved in the pathogenesis. This occurs due to tissue damage [1] and deregulation of host immune reactions in response to the pathogenic fungi [2,3]. Direct and indirect interactions between fungi and bacteria may lead to both antagonistic and synergistic effects on survival [2,4,5,6]. In addition, interrelations between fungi and bacteria in insect hosts may be mediated by complex environmental factors, such as temperature, chemicals, or parasitoids, that have an influence on the outcome of the disease [7,8]. However, these immune-ecological studies are just beginning to develop.

Temperature is one of the crucial factors that influences the development of mycoses and bacterioses in insects. Temperature acts on both microorganism growth and on insect immune and behavioral reactions [9,10,11,12,13,14]. Entomopathogenic ascomycetes usually have optimal growth between 20–30 °C [15]. In contrast, many bacteria that are associated with terrestrial insects exhibit more active growth between 28–37 °C [16,17]. In many cases, host cellular and humoral antifungal reactions and resistance to fungi are increased with a short or prolonged elevation of temperature [18,19,20,21,22,23,24]; however, cold stresses may also activate antifungal systems [18]. Insect antibacterial responses are also dependent on environmental temperatures [25,26,27] and elevated temperature often promotes bacterial infection [16,17]. There are examples of increased antibacterial responses in insects under short-term or prolonged exposure to low temperatures [16,26,28,29]. Importantly, in a state of cold diapause, cellular immunity continues to work [30] and changes in microbiome composition, immune response and susceptibility to fungal and bacterial infections may also occur [27,31]. It is likely that the outcome of complex infections may be shifted toward mycoses under low temperatures and toward bacterioses under high temperatures. However, the changes in immune response and microbiota composition during complex infections under different temperature conditions are insufficiently understood.

Various antimicrobial peptides (AMPs) of insects have key roles in both antibacterial and antifungal responses [32]. Some AMPs, such as gallerimycin and galiomycin, which are regulated by the Toll immune signaling pathway, exhibit activities against filamentous fungi, but not against bacteria [33,34]. Many AMPs (e.g., cecropins, gloverins, lysozymes) synthesized via the IMD and Toll pathways have a broad spectrum of activities predominantly against gram-positive and gram-negative bacteria as well as against fungi [35,36]. It is likely that AMPs control the proliferation of bacteria during the development of mycoses. In fact, the level of AMP gene expression clearly responds to changes in the microbial community during fungal infections [3]. Moreover, AMP gene expression is dependent on temperature [23,29].

The expression of apoptosis, reactive oxygen species (ROS) and stress-related genes may be crucial in the development of infections caused by entomopathogenic fungi and concomitant bacteria. In particular, a key regulator of programmed cell death, inhibitor of apoptosis (IAP), has paramount physiological importance, including in the antifungal response as was recently shown by Zhang and coworkers [37]. RNAi-mediated knockdown of the IAP homologue in locusts led to a decrease in the total hemocyte count, a degeneration of the gut, a shift in the microbiota, and increased susceptibility to fungal infection. In addition, IAP is involved in immunity to bacterial infections, as shown for *Drosophila* [38]. The generation of ROS has a large impact in reactions against different pathogens [39], as well as in maintaining microbial homeostasis, especially in the insect gut [40,41,42]. The main source of ROS in insect hemolymph is the prophenoloxidase cascade. As a result of its activation, ROS (primarily semi-quinone radicals and H_2_O_2_) are formed [43,44,45]. In the gut, fat body and in other tissues, the formation of ROS occurs with the participation of members of the NADPH oxidase (NOX) family, such as dual oxidases (DUOX). The enzyme generates superoxide and H_2_O_2_, which are powerful oxidants that exhibit microbicidal activity [40]. Both fungal and bacterial infections led to changes in DUOX activity in the gut and hemocoel tissues [2,3,42]. RNAi knockdown of the DUOX system caused a decrease in ROS and uncontrolled proliferation of bacteria [42]. Heat shock proteins (HSPs) have functions in protein folding and unfolding, and participate in immune signaling pathways and other processes [46]. HSPs are important stress markers, which sense different thermal actions, diapause formation [47] and infections [12].

It is important to note that entomopathogenic fungi produce various secondary metabolites and enzymes (oosporeins, destruxins, different proteinases, AMPs) for inhibiting both host immune responses and competitive microorganisms [36,48,49]. The set of enzymes and secondary metabolites present is significantly different between fungal species and depends on host and habitat specificity. As a rule, generalist species have a broader spectrum of metabolites compared to species with restricted host ranges [50,51,52].

The ascomycete *Cordyceps militaris* is characterized by a restricted host and habitat range and has a highly reduced number of genes involved in secondary metabolism and the synthesis of proteases compared to generalists such as *Metarhizium robertsii* and *Beauveria bassiana* [50,53]. This fungus mainly infects forest lepidopterans (Lepidoptera, Macroheterocera) in the larval and pupal stages which are located in the soil, forest flour and fallen wood [54,55,56]. Previously, natural infections of insects with *C. militaris* were studied insufficiently. It is known that larvae and pupae could be infected by topical application with ascospores or conidia in a laboratory [22,57]. However this method is difficult to reproduce, and the outcomes strongly depend on the physiological state of the host [22]. In contrast, injection of lepidopteran larvae and pupae with blastospores or conidia has led to more stable development of the mycosis [55,58,59]. Importantly, spontaneous bacterial infections have been constantly documented after infection of *C. militaris* with insects in laboratory conditions [22,58,59]. Therefore, *C. militaris* is a convenient model to study fungal–bacteria interactions in insects. The optimal temperature for mycelial growth of *C. militaris* palearctic isolates is approximately 20 °C [22,60]. In a previous study [22], we showed in a model insect, the wax moth *Galleria mellonella,* that larvae in a state of facultative diapause induced by a low temperature (15 °C) are most susceptible to the fungus. Mycosis successfully developed after injection with *C. militaris* blastospores at 15 °C. By contrast, at 25 °C (active state), larvae were able to overcome the infection and complete metamorphosis, although, the infection may persist in pupae and adults and could still be activated by a low temperature. Activation of the antifungal response (encapsulation and phenoloxidase activity) in response to *C. militaris* infection was observed in wax moth larvae at 25 °C, while inhibition of these parameters occurred at 15 °C. We suggested that *C. militaris* uses fewer universal tools for evasion and inhibition of host immunity compared to generalists fungi, such as *Metarhizium* and *Beauveria,* that induce prolonged mycosis development, persist in hosts and have a specialization in killing dormant insects with reduced immune activity [22,59]. Moreover, because we registered spontaneous bacterioses in the wax moths post injection of *C. militaris* blastospores and conidia, we hypothesized that *C. militaris* has poorly developed mechanisms for manipulating the host microbiota and requires a low temperature for its normal development.

In the present study we investigated the microbial communities of the wax moth larvae hemolymph and midgut, as well as the expression of AMP, apoptosis, ROS and stress-related genes in the wax moth midgut and fat body after injection with *C. militaris* and incubation under two temperatures, 15 °C (state of facultative diapause) and 25 °C (active state). We found significant changes in these parameters in response to infection at different temperatures, which support the hypothesis mentioned above.

## 2. Material and Methods

### 2.1. Fungi and Insects

*C. militaris* isolate CNAp (GenBank No MF073255.1), from the microorganism collection of the Institute of Systematics and Ecology of Animals SB RAS, was used in this work. Conidia had been stored at −80 °C since 2015. For infections, conidia were cultivated on Sabouraud dextrose agar with yeast extract (2.5 g/L) (SDAY) for 22 days at 23 °C and a photoperiod of 8:16 (light:dark). Conidia were suspended in saline (0.9% NaCl) without any detergents and filtered through a sterile cloth to remove mycelial clumps. Concentrations of conidia were determined using a Neubauer hemocytometer. A Siberian line of wax moth larvae was maintained on artificial media as described previously [61]. Larvae of the sixth instar were used in experiments.

### 2.2. Procedures for Infection and Bioassays

Larvae were injected with 4 μL of a suspension containing 1250, 2500 or 5000 conidia. Control larvae were injected with saline. Punctures were made between the sixth and seventh abdominal segments using a microinjector with an insulin syringe. The needle was sterilized with 96% ethanol before each injection. Infected and control larvae were placed at two constant temperatures (25 °C and 15 °C) immediately after injection. Larvae were maintained in 90 mm glass Petri dishes (12 larvae per dish) with artificial media (3 g per one Petri dish) in the dark. Temperature in the Petri dishes at such an insect density corresponded with environmental temperatures. Ventilation of the Petri dishes and registration of mortality was conducted every day over 10 days. To determine the causes of death, cadavers were placed on moist filter paper in the Petri dishes and maintained at the temperatures indicated above. Three replicates (one replicate = 12 larvae) were used to assay mortality after injection with each dose, and the whole experiment was repeated twice. 

For detection of hyphal bodies and bacteria in the hemolymph of infected larvae, we used gradient centrifugation of the hemolymph in Percol followed by electron transmission microscopy as described previously [59]. To determine the yield of conidia on the larvae, the cadavers were incubated for 30 days in moist chambers at 25 °C and 15 °C. Then, each cadaver was placed in a tube with 20 mL of a 0.1% water-Tween 20 solution and vortexed for 3 min until the mycelia and conidia were completely washed off. Conidia were counted using a hemocytometer and the concentrations were calculated for each cadaver.

### 2.3. Bacterial Colony Forming Unit (CFU) Counts

At 96 h after injection with a dose of 2500 conidia per larvae, control and infected insects were surface sterilized by 3% H_2_O_2_ and 70% ethanol. Forty five μL of hemolymph from three larvae were placed in 100 μL of 150 mM cool NaCl and immediately homogenized using an ultrasonic homogenizer (Sonopuls, Bandelin electronic GmbH & Co. KG, Berlin, Germany). Midguts were pooled in the same NaCl (one sample = three larvae) and homogenized by the same technique. Samples were diluted with the same NaCl by 10, 100, and 1000-fold and a 100 μL aliquot was plated on media (Bile esculin azide agar for enterococci and Endo agar for enterobacteria) in 90 mm Petri dishes. The cultures were incubated for 2 days at 35 °C and then CFUs were counted. CFU counts were calculated for each midgut or 10 μL of hemolymph. A total of 5–6 samples from each treatment were used for analysis.

### 2.4. Analysis of Bacterial Communities

At 96 h post-treatment, infected (2500 conidia) and control larvae were surface-sterilized by 3% H_2_O_2_ and 70% ethanol and dissected. Midguts with content were isolated and frozen in liquid nitrogen (one sample = 5 midguts). In addition, decomposed cadavers (6–7 d post infection) were analyzed. Whole cadavers were frozen in liquid nitrogen (one sample = 3 whole bodies). Three biological replicates from each treatment were used.

DNA was isolated using a DNeasy PowerSoil DNA Isolation Kit (Qiagen, Hilden, Germany) according to the manufacturer’s protocol. The 16S rRNA region was amplified with the primer pair V3–V4 combined with Illumina adapter sequences [62]. PCR amplification was performed as described previously [63]. A total of 200 ng of PCR product from each sample was pooled together and purified using a MinElute Gel Extraction Kit (Qiagen, Hilden, Germany). The obtained libraries were sequenced with 2 × 300 bp paired-end reagents on a MiSeq (Illumina Inc., San-Diego, California, USA) in the SB RAS Genomics Core Facility (ICBFM SB RAS, Novosibirsk, Russia). The sequencing data reported in this study were submitted to GenBank under the study accession PRJNA650299.

Raw sequences were analyzed with the UPARSE pipeline [64] using Usearch v11.0. The UPARSE pipeline included a merging of paired reads, read quality filtering, length trimming, merging of identical reads (dereplication), discarding singleton reads, removing chimeras, and operational taxonomic unit (OTU) clustering using the UPARSE-OTU algorithm. The OTU sequences were assigned a taxonomy using the SINTAX [65] and 16S RDP training set v.16 [66]. The final dataset included 384,875 reads (mean ± SE = 20,532 ± 630 per midgut sample and 46,162 ± 1453 per cadaver sample, see Dataset). All rarefaction curves showed a trend of approaching the saturation plateau (Figure S1), which indicated a reasonable volume of the sequenced reads.

### 2.5. Gene Expression 

Wax moth larvae at 96 h after injection (2500 conidia) were dissected on ice cold PBS and midguts without contents and fat bodies were collected. Midguts content were removed by eye forceps. Midguts from ten larvae or fat bodies from five larvae were pooled in each sample. A total of 5–6 samples (biological replicates) from each treatment were used for analysis. The tissues were frozen in liquid nitrogen and stored at −80 °C. Samples were lyophilized at −65 °C, 400 mtorr for 15 h and disrupted in liquid nitrogen using micropestles just before RNA isolation. The tissues were homogenized in QIAzol Lysis Reagent (Qiagen, Hilden, Germany) and RNA was isolated according to the manufacturer’s instructions. Quantity and quality of the total DNA were estimated by NanoDrop NanoVue Plus (GE Healthcare, Chicago, Illinois, USA). Each sample was normalized to a concentration of 1.5 µg/µL and treated by RQ1 RNase-free DNase (Promega, Madison, WI, USA). RNA was converted to cDNA using 6 μg DNA-free RNA, 3 µL 100 nM random nanomers and 4 µL RevertedAid^TM^ M-MuLV Reverse Transcriptase (Fermentas, Vilnius, Lithuania).

qPCR was carried out using HS-qPCR SYBR Blue (2×) mix (BioLabMix, Novosibirsk, Russia) with a CFX96 Touch (Bio-Rad Laboratories, Inc., Hercules, CA, USA). qPCR was performed in triplicate under the following conditions: 95 °C for 3 min, and 40 cycles of 15 s at 94 °C and 30 s at 60/62/64 °C (depending on the primer Tm), followed by melt curves (70–90 °C). Gene expression was estimated by the ΔΔCq protocol with Bio-Rad CFX Manager (Bio-Rad, Laboratories, Inc., Hercules, CA, USA). The following *G. mellonella* genes were used as references: translation elongation factor 1-alpha 1 (eEF1α1) and the subunit of DNA-directed RNA polymerase II. The expression dynamics of the following ten genes of interest were studied: antimicrobial peptides gallerimycin, galiomycin, gloverin, cecropin-like and lysozyme-like, apoptosis-related IAP, the ROS-related NOX-DUOX domain and heat shock proteins Hsp70 and Hsp90. These genes and primer sequences were from the work of Lange and coauthors [67] and Melo and coauthors [68] or designed by us (Table S1). Primer properties were estimated by IDT OligoAnalyser 3.1 (http://eu.idtdna.com/calc/analyzer). Primers were synthesized by Biosintez, Koltsovo, Russia.

### 2.6. In Vitro Interaction between Fungi and Bacteria 

For the interaction studies, we used the predominant cultivable bacteria previously isolated from *G. mellonella* midgut, *Enterococcus faecalis* and *Enterobacter* sp. [7] In addition to *C. militaris*, the fungi *M. robertsii* (strain MB-1) and *B. bassiana* (strain Sar-31) from the microorganism collection of the Institute of Systematics and Ecology of Animals SB RAS were used as positive controls. Bacteria were cultivated on nutrient agar (Himedia, Mumbai, India) and fungi were cultivated on SDAY media. One-day-old plugs of bacteria (8 mm) or plugs of nutrient agar (control) were placed on freshly plated cultures of fungi in 90 mm Petri dishes. Zones of mycelial growth inhibition were measured at 4 days of incubation at 25 °C. Similarly, four-day-old plugs of fungi were placed on freshly plated bacterial cultures. Zones of growth inhibition were estimated on the first and second day of incubation at 25 °C. Radial mycelial growth on cultures of bacteria was recorded over 24 days. As a control, measurements of mycelial growth on bacteria-free nutrient agar were conducted. Three replicates were used in each treatment.

### 2.7. Statistics

Differences in mortality dynamics were analyzed by a log rank test followed by Holm–Sidak adjustment. The χ^2^ criterion was applied in estimating the portion of sporulated and decomposed larvae. Other data were checked for normality of distribution using the Shapiro–Wilk W test. Conidia yields from cadavers had a normal distribution and were analyzed by a Student *t*-test. Data from CFU counts, OTU abundances, diversity indexes and gene expression had abnormal distributions and were analyzed by a nonparametric analogue of the two-way ANOVA, namely, the Scheirer–Ray–Hare test [69], followed by Dunn’s post hoc test. Data from the antagonistic interactions between fungi and bacteria in vitro were analyzed by the Kruskal–Wallis test with Dunn’s post hoc test.

## 3. Results

### 3.1. Bioassays

**Figure 1:**
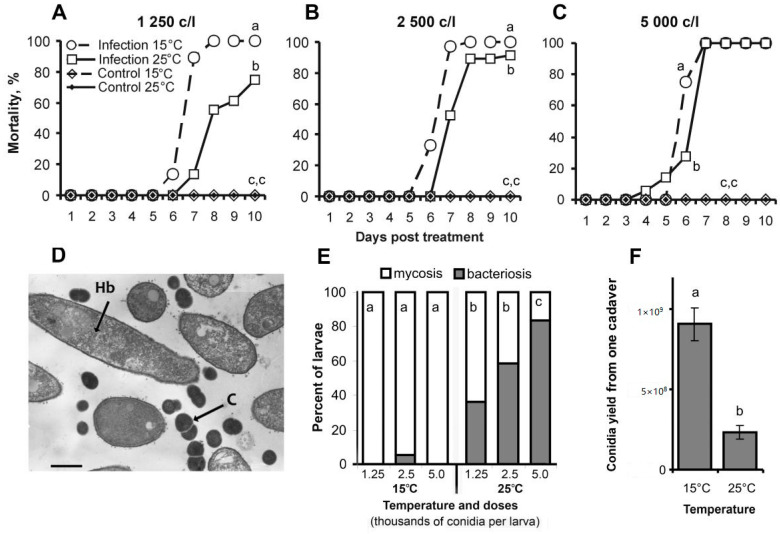
Mortality dynamics and outcome of infection in wax moth larvae after injection with *C. militaris* conidia and subsequent incubation at 15 °C and 25 °C. (**A**–**C**)—mortality dynamics after injection of larvae with 1250, 2500 and 5000 conidia per larva (c/L). Different letters indicate significant differences determined by log rank test (χ^2^ > 9.6, df = 1, *p* < 0.002). (**D**)—*C. militaris* hyphal bodies (Hb) and cocci (C) in wax moth hemolymph at 5 days after injection with the fungus. Scale bar, 1 µm. (**E**)—portion of mummified and decomposed larvae during the development of mycoses at different temperatures. Different letters indicate significant differences (χ^2^ > 8.7, df = 1, *p* < 0.003). (**F**) –*C. militaris* conidial yield on mummified cadavers at 15 °C and 25 °C. Different letters indicate significant differences determined by *t*-tests (*t* = 6.1, df = 8, *p* < 0.001).

Mortality of larvae injected with *C. militaris* conidia began at 5–7 days post injection and reached 80–100% after 7–10 days, depending on the dose and temperature (Figure 1A–C). More rapid mortality of larvae at 15 °C compared to 25 °C was observed following injection of all doses (log rank test, χ^2^ > 9.6, df = 1, *p* < 0.002). No mortality was registered for larvae injected with saline. Notably, 9–20% of insects infected with low and intermediate doses and maintained at 25 °C were able to survive and complete metamorphosis.

**Figure 2:**
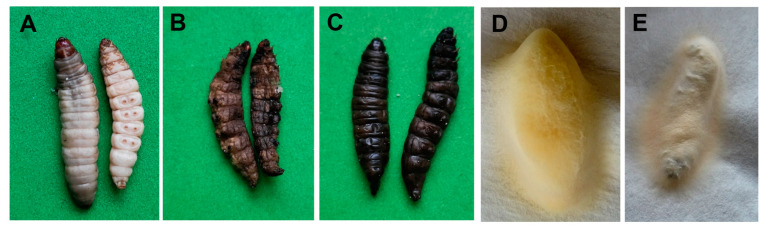
Phenotypes of larvae that died after injection with *C. militaris* conidia. (**A**)—mummification, (**B**)—defective mummies, (**C**)—bacterial decomposition, (**D**)—conidiation at 15 °C, (**E**)—conidiation at 25 °C.

Microscopy observations showed the simultaneous presence of hyphal bodies and cocci in the hemolymph of infected insects maintained at 25 °C (Figure 1D), however, these cocci were not observed in the hemolymph at 15 °C. At 15 °C, mycosis led to the formation of mummified cadavers (94–100%) after all treatment doses (Figure 1E and Figure 2A). However, at 25 °C, we documented a large number of bacterially decomposed insects (χ^2^ > 8.7, df = 1, *p* < 0.003 compared to 15 °C). The bacterioses were identified by symptoms of darkening and liquefaction of the larvae for several hours after death (Figure 2C). The increase in frequency of bacterioses at 25 °C was dose-dependent and increased from 36% after injection with the lowest dose and to 83% after injection with the highest dose (Figure 1E). Notably, we registered the formation of abnormally dark mummies in these experiments (Figure 2B). The percent of abnormal mummies was 52% at 25 °C and only 12% at 15 °C (χ^2^ = 8.3, df = 1, *p* = 0.004). Moreover, the production of conidia on mummified cadavers at 25 °C decreased 2.5-fold compared to 15 °C (*t* = 6.1, df = 8, *p* < 0.001, Figure 1F and Figure 2D,E). Thus, insects were less susceptible to *C. militaris* infection at 25 °C, but they were more predisposed to spontaneous bacterial infections compared to those incubated at 15 °C.

### 3.2. CFU Counts in the Hemolymph and Midgut

**Figure 3:**
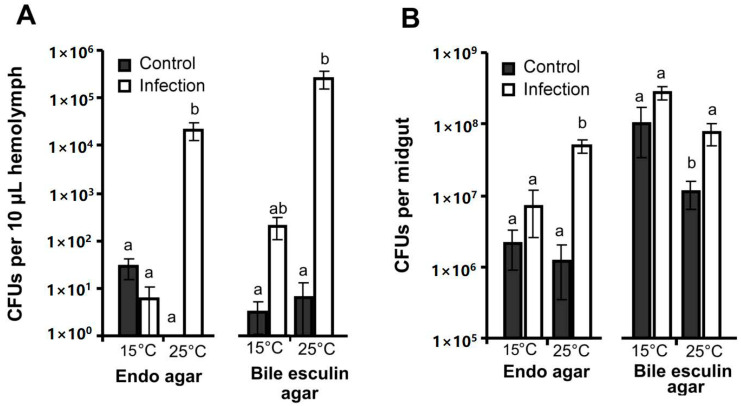
CFU counts in the hemolymph (**A**) and midgut (**B**) of wax moth larvae at 96 h post injection of *С. militaris* (2500 conidia per larva) with subsequent incubation at 15 °C and 25 °C. Selective media for enterobacteria (Endo agar) and enterococci (Bile esculin agar) was used. Different letters show significant differences within the specified media and tissue (Dunn’s test, *p* < 0.05).

In the hemolymph of control larvae, we registered single colonies of enterococci and enterobacteria at both temperatures (Figure 3A). At 15 °C, fungal infection did not lead to significant changes in the CFU count (Dunn’s test, *p* > 0.17 compared to controls). In contrast, CFU counts of both enterobacteria and enterococci increased in the hemolymph by 39,000–54,000-fold at 25 °C in response to *С. militaris* infection (*p* < 0.002 compared to controls). A significant interaction between factors (mycosis × temperature) was found for enterobacteria (H_1,19_ = 9.4, *p* = 0.002). However, this interaction was not found for enterococci (H_1,19_ = 1.5, *p* = 0.23) because there was still a slight increase in these bacteria at 15 °C in response to *С. militaris* infection.

In the midgut, we observed an elevation in the enterobacteria and enterococci CFU counts in response to fungal infection at both temperatures (enterobacteria, H_1,19_ = 4.2, *p* = 0.04; enterococci, H_1,19_ = 8.5, *p* = 0.004). However, the post hoc tests showed significant elevation only at 25 °C (4–7-fold relative to controls, Dunn’s test, *p* < 0.04, Figure 3B). Effects of temperature on CFU counts were not significant, however, a trend toward increased enterococci was observed at 15 °C compared to 25 °C (H_1,23_ = 2.7, *p* = 0.09). Notably, uninfected larvae maintained at 15 °C were characterized by the highest enterococci CFU counts compared to larvae maintained at 25 °C (*p* = 0.03).

### 3.3. Bacterial Communities in Midguts and Cadavers 

**Figure 4:**
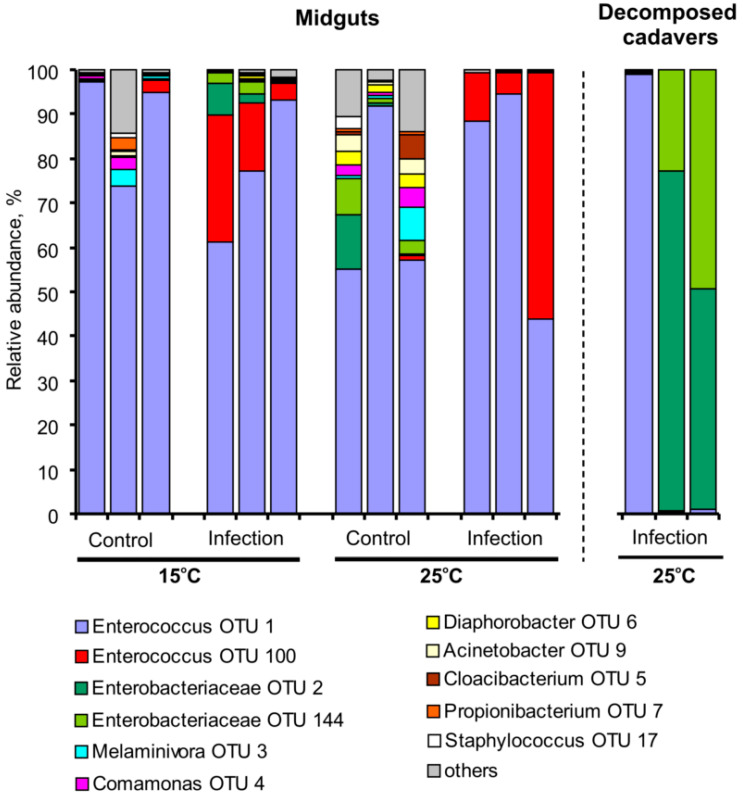
Bacterial communities (16S rRNA) in the midguts of wax moth larvae during the development of *С. militaris* infection at different temperatures and the communities in the cadavers that decomposed after the infection. Midgut communities were analyzed at 96 h after injection with a dose of 2500 conidia per larva. Decomposed cadavers were analyzed at 6–7 days post injection. Each treatment represents 3 replicates.

In the midgut, we registered 168 OTUs (37 ± 5.2 OTUs per sample) with a predominance of two *Enterococcus* OTUs (Figure 4). A BLAST search against sequences in GenBank showed strong similarity with *Enterococcus faecalis* (OTU 1, 100% similarity) and *E. lemanii* (OTU 100, 99.53% similarity). Temperature did not have a significant impact on the relative abundance of different groups and diversity indexes (H_1,11_ < 0.4, *p* > 0.52, Figure S2). However, trends toward increased diversity indexes in warm conditions were observed for uninfected larvae (Dunn’s test, *p* > 0.08, Figure S2). Fungal infection led to a significant decrease in OTU counts and the Chao1 index (H_1,11_ > 4.7, *p* < 0.03), as well as to shifts in community structure. Under both temperatures, *C. militaris* infection caused a partial displacement of *E. faecalis* by *E. lemanii* (effect of the infection: H_1,11_ = 8.3, *p* = 0.004). In addition, a decrease in the abundance of the subdominant bacteria *Acinetobacter, Melaminivora, Comamonas*, and *Diaphorobacter* was revealed under the influence of the fungal infection (H_1,11_ > 5.0, *p* < 0.024). These effects were more evident at 25 °C (Dunn’s test, *p* < 0.013) compared to 15 °C (Dunn’s test, *p* > 0.17).

In bacterially decomposed cadavers, we registered the lowest bacterial diversity (OTU count, 10 ± 1.9; Chao1, 13 ± 2.6; Shannon, 0.47 ± 0.14). In the cadavers, either the enterococci *E. faecalis* or *Enterobacter* sp. prevailed (Figure 4). Enterobacteriaceae were represented by two predominant OTUs that were also detected in the midgut. One of them, OTU 2, was close in identity to *Enterobacter* sp. (99.53% similarity) which was previously isolated from the midgut of same line of *G. mellonella* [7]. The other, OTU 144, was close to *Cronobacter sakazakii* (99.77% similarity).

### 3.4. AMP Gene Expression

**Figure 5:**
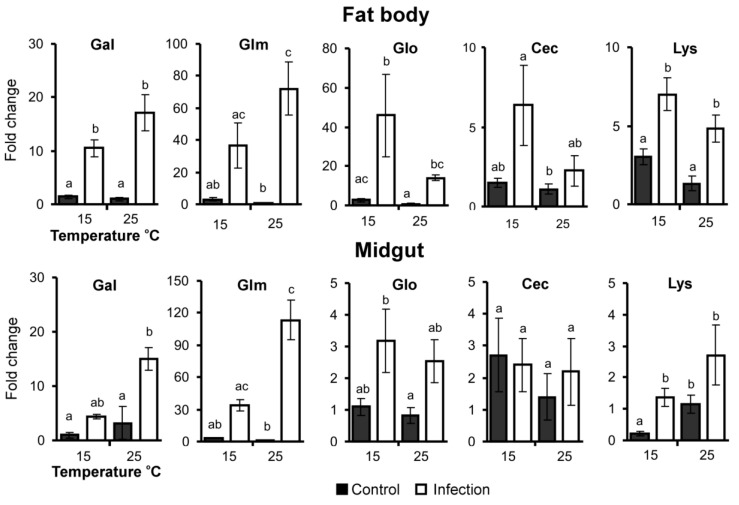
Relative expression of AMP genes in the fat body and midgut of wax moth larvae at 96 h after injection with *С. militaris* (2500 conidia per larva) and subsequent incubation at 15 °C and 25 °C. Data were normalized to the expression of two reference genes, eEF1a and RBP11. The *Y*-axis shows the fold change relative to uninfected larvae maintained at 25 °C. **Gal**—galiomycin, **Glm**—gallerimycin, **Glo**—gloverin, **Cec**—cecropin-like, **Lys**—lysozyme-like. Different letters indicate significant differences between treatments (Dunn’s test, *p* < 0.05).

We observed a significant upregulation in the expression of the studied AMP genes (except for cecropin) in both the fat body and the midgut under the influence of fungal infection (Figure 5, Table 1). Temperature had a significant impact on the expression of cecropin and lysozyme only. Overall, we observed a stronger expression of antifungal peptide genes in response to infection at 25 °C compared to 15 °C. In contrast, antibacterial peptide genes trended toward higher expression at 15 °C compared to 25 °C. For example, expression of the antifungal peptide gene gallerimycin in the fat body was increased by 77-fold at 25 °C, but only by 12-fold at 15 °C compared to uninfected insects (Dunn’s test, *p* < 0.0005 and *p* = 0.10, respectively). The galiomycin gene in the fat body was upregulated by 18-fold at 25 °C but only 8-fold at 15 °C (*p* = 0.001 and *p* = 0.04 compared to controls, respectively). Gallerimycin and galiomycin gene expression followed the same pattern in the midgut (Figure 5, Table 1).

Unlike the antifungal peptides, expression of the antibacterial peptide gloverin in the fat body in response to fungal infection increased by 55-fold at 15 °C (*p* = 0.005 compared to control) and 17-fold at 25 °C (*p* = 0.01 compared to control). For the cecropin and lysozyme genes, we observed increased expression in the fat body at 15 °C compared to 25 °C (effect of temperature, H_1,19_ = 3.9, *p* = 0.05 and H_1,23_ = 3.2, *p* = 0.07, respectively), and more active expression in response to fungal infection was also observed at low temperature (Figure 5). Changes in the expression of the gloverin, cecropin and lysozyme peptide genes in the midgut were less than in the fat body. The gloverin gene was upregulated by 2.8-3-fold in the midgut in response to fungal infection (H_1.19_ = 6.2, *p* = 0.01), independent of temperature. Expression of cecropin in the midgut was not significantly changed in response temperature or fungal infection. Expression of the lysozyme gene in the midgut was decreased at 15 °C compared to 25 °C (H_1,23_ = 4.6, *p* = 0.03); however, there was a significant upregulation in response to *C. militaris* infection, which occurred only at 15 °C (6-fold, *p* = 0.004 compared to control) and not at 25 °C (2-fold, *p* = 0.15 compared to control).

### 3.5. Apoptosis, ROS and Stress-Related Gene Expression 

**Figure 6:**
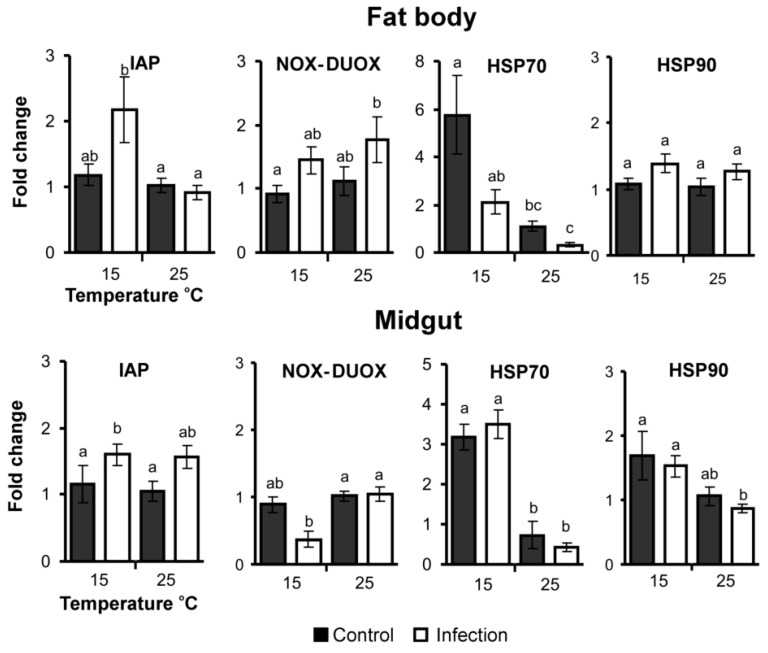
Relative expression of apoptosis, ROS and stress-related genes in the fat body and midgut of wax moth larvae at 96 h after injection with *С. militaris* (2500 conidia per larva) and subsequent incubation at 15 °C and 25 °C. Data were normalized to the expression of two reference genes, eEF1a and RBP11. The *Y*-axis shows the fold change relative to uninfected larvae maintained at 25 °C. Different letters indicate significant differences between treatments (Dunn’s test, *p* < 0.05).

Expression of the IAP gene in the fat body was temperature dependent (Figure 6, Table 2). The gene was upregulated in the fat body in response to fungal infection only at low temperature (Dunn’s test, *p* = 0.07 compared to control at 15 °C and *p*< 0.01 compared to other treatments). At 25 °C, expression of this gene in response to infection was not changed compared to the control (*p* = 0.50). In the midgut, regulation of the IAP gene was not caused by temperature (Table 2), but only by the infection (effect of fungus: H_1,23_ = 7.7, *p* = 0.006). Significant upregulation in response to *C. militaris* was also registered only at 15 °C (*p* = 0.04 compared to control).

NOX-DUOX domain gene expression was slightly (1.6-fold) upregulated in the fat body in response to fungal infection at both temperatures (effect of infection: H_1,23_ = 4.2, *p* = 0.04), but the effect of temperature was not significant (Table 2). Scheirer–Ray–Hare test showed a downregulation of this gene in the midgut under the influence of a low temperature (H_1,23_ = 6.16, *p* = 0.01), however strong downregulation (>2.4-fold) was observed during mycosis development at 15 °C only (*p* = 0.08 compared to control at 15 °C and *p* < 0.01 compared to other treatments). 

HSP70 gene expression in the fat body was increased at a low temperature (Figure 6, Table 2) and fungal infection downregulated its expression at both temperatures (effect of fungus: H_1,23_ = 4.6, *p* = 0.03). In the midgut, upregulation of HSP70 was also observed at 15 °C (H_1,23_ = 13.7, *p* < 0.001) but fungal infection had no significant effect. The HSP90 gene was slightly and insignificantly upregulated in the fat body in response to infection and independent of temperature. Its expression in the midgut was increased under the influence of low temperature (H_1,23_ = 8.7, *p* = 0.003) but fungal infection had no significant effect.

### 3.6. Interaction between Fungi and Bacteria In Vitro

**Figure 7:**
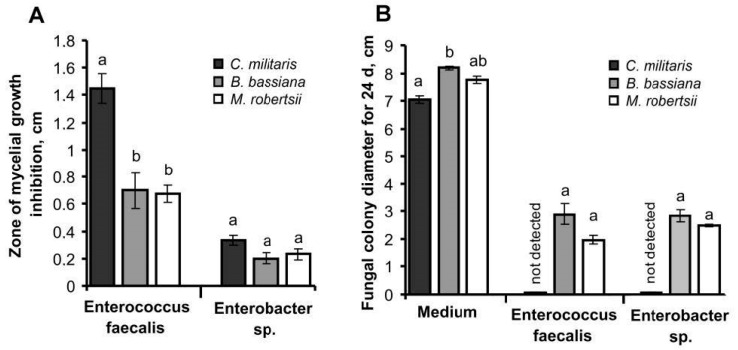
Inhibition of fungi by *Enterococcus faecalis* and *Enterobacter sp*. in vitro. (**A**)—zone of mycelial growth inhibition by bacteria on SDAY medium. (**B**)—radial growth of fungi on nutrient agar and this medium with lawns of the bacteria. Different letters indicate significant differences between treatments (Dunn’s test, *p* < 0.05).

We showed that *E. faecalis* and *Enterobacter* inhibited *C. militaris* more strongly than *M. robertsii* and *B. bassiana*. In particular, *E. faecalis* inhibited *C. militaris* mycelial growth on SDAY medium by 2–2.2-fold more than *M. robertsii* or *B. bassiana* growth (Dunn’s test, *p* < 0.012, Figure 7). *Enterobacter* sp. also inhibited *C. militaris* growth more strongly than *M. robertsii* and *B. bassiana*, but the differences were only marginally significant (*p* = 0.06–0.10). None of these fungi inhibited bacterial growth on nutrient agar. However, *M. robertsii* and *B. bassiana* were able to grow on cultures of both bacteria (Figure 7). In contrast, *C. militaris* was not able to grow on cultures of *E. faecalis* or *Enterobacter* sp.

## 4. Discussion

**Figure 8:**
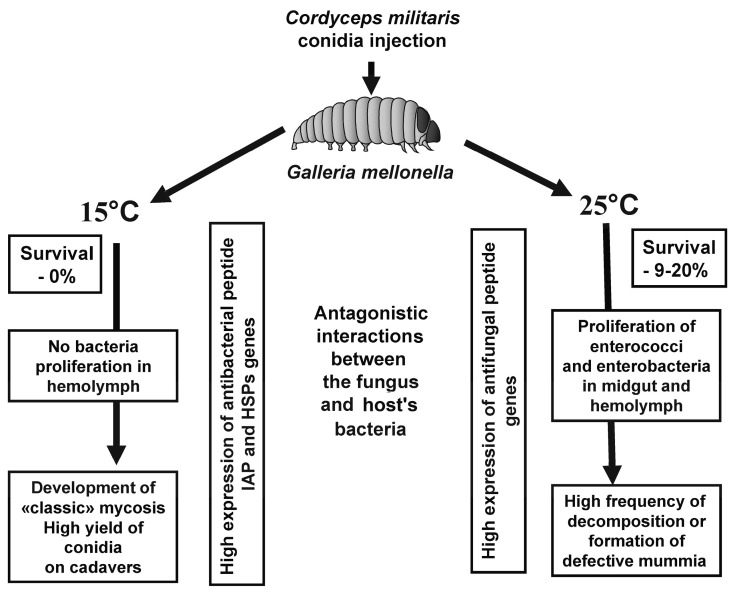
Outline of the interactions between *C. militaris* and bacterial associates in wax moth larvae under different temperature conditions.

The development of mycoses in insects is not restricted to fungal monoinfections, and bacterial commensals and pathogens are also involved in this process [1,2,5]. We show that bacterial involvement in fungal pathogenesis and its outcome is dependent on environmental conditions, particularly temperature (Figure 8). The development of *C. militaris* in wax moth larvae was faster and more successful at 15 °C compared to 25 °C. At 25 °C, fungal virulence was decreased however a high frequency of spontaneous bacteriosis was observed, which was caused by the proliferation of enterococci and enterobacteria in the hemolymph. We also showed that *C. militaris* is a weak competitor of bacteria compared to generalist fungi such as *M. robertsii* and *B. bassiana*. This is consistent with the more specific conditions for cultivation required by *C. militaris* in vivo or in vitro [70]. Occurrence of bacterioses after topical infection or injection of *C. militaris* in insects has been documented previously [22,55,58,71]. We suggest that *C. militaris* has a limited ability to suppress host commensal bacteria, as the fungus is associated with narrow environmental requirements, including a specific temperature range [22]. Low temperatures (~15 °C) limit the active influence of bacteria on fungal pathogenesis. This may be explained by the fact that low temperatures are suboptimal for the proliferation of many bacteria. In addition, we showed a stronger antibacterial response in the host during *C. militaris* development under low temperature conditions.

Consistent with our study of the midgut microbiome, the predominant bacteria in the gut of healthy wax moths are *Enterococcus* species [72,73]. In different pathological states (e.g., toxicosis caused by *Bacillus thuringiensis* or envenomation with pаrаsitoids), a shift in the microbiome structure toward Enterobacteriaceae prevalence occurred in the wax moth gut [7,74]. However, we observed another effect in the present study, the replacement of one *Enterococcus* species with another under the influence of a fungal infection. Change in the dominance between different *Enterococcus* species was also documented previously after injection of wax moth larvae with *C. militaris* blastospores (unpublished [75]). The mechanism of this restructuring is not clear and is likely associated with the selective action of fungal metabolites on different species of enterococci. For example, significant changes in the mouse gut microbiome were observed after feeding mice a major metabolite of *C. militaris* cordycepin [76]. Gamage and coworkers [77] showed that *С. militaris* water extracts exhibited different levels of inhibition of various gram-positive and gram-negative bacteria.

We observed an increase in bacterial CFU counts in the midgut during the development of *C. militaris* infection. This confirmed previous work performed on adult mosquitos following topical infection with *Beauveria* and *Isaria* species [2,3], as well as work on Colorado potato beetle larvae after topical treatment with *Metarhiziun robertsii* [78]. These enhancements may be caused by a disturbance in feeding, gut peristalsis or by a deregulation in immune reactions during mycosis development. It should be noted that significant elevations in enterococci and enterobacteria loads in response to *C. militaris* infection were observed only in warm (25 °C) conditions and not in cold (15 °C) conditions.

In the hemolymph of uninfected larvae, we observed single colonies of enterococci and enterobacteria. Dramatic (39–54-thousand-fold) elevations in the CFU counts of these bacteria in the hemolymph during fungal infection were observed only in warm conditions (25 °C). It should be noted that the enterococci are a prevalent group of bacteria in wax moth integuments and enterobacteria are also present in these tissues [73,79]. However, it is hardly possible that the observed septicemia was the result of an influx through a cuticle puncture since bacterial-induced death began at five days post injection and occurred simultaneously with death due to mycosis. Moreover, the frequency of spontaneous bacterioses at 25 °C was positively correlated with the dose of *C. militaris* conidia. The source of bacterial penetration into the hemolymph could be the gut or other organs such as the trachea or excretory organs, the biome of which has not been studied in the wax moth. It is interesting to note that the occurrence of septicemia was less common after injection of wax moth larvae with conidia of the generalist fungi *Metarhizium* or *Beauveria*. For example, injection of the larvae with *B. bassiana* and *M. robertsii* at doses of 2500 conidia per larvae and subsequent incubation at 25 °C did not lead to bacterial decomposition and all cadavers were mummified and overgrown with these fungi (Figure S3). Fan and coauthors [48] showed that at the final stages of mycoses, *B. bassiana* suppresses the proliferation of bacteria in the host through the production of secondary metabolites such as oosporeins. However, compared to *Beauveria* and *Metarhizium* species, *C. militaris* has fewer genes involved in secondary metabolism [50,53]. It is likely that the combination of less developed mechanisms for the suppression of bacteria and harsher tools for host tissue destruction caused the septicemia during *C. militaris* infection. In particular, we recently showed that *C. militaris* infection led to necrotic death of hemocytes and a strong elevation in dopamine and ROS in wax moth larvae, which were not observed after *M. robertsii* infection [59].

The development and outcome of the fungal infections can also be mediated by differences in host immune responses at 15 °C and 25 °C. Antifungal peptides (gallerimycin and galiomycin) more actively responded to *C. militaris* infection at a higher temperature. This is consistent with previous investigations in which we showed a stronger elevation in phenoloxidase and encapsulation levels in wax moths in response to *C. militaris* infection at 25 °C compared to 15 °C [22], and this correlated with a greater survival of the infected insects at 25 °C. It is interesting that the antifungal peptide genes were actively expressed at 25 °C, not only in the fat body but also in the midgut. This may be due to a systemic immune response or an attack of lateral midgut tissues by the fungus. Unlike the antifungal response, the expression of antibacterial peptide genes (gloverin, cecropin, lysozyme) was more active in the fat body at 15 °C, which correlated with the absence of elevated CFUs and bacterial decomposition at this temperature. It was previously shown that a short exposure of *G. mellonella* to low temperatures led to an increase in AMP expression in response to *B. thuringiensis* infection [28]. Similar exposure led to enhanced AMP expression in *Ostrinia furnacalis* in the absence of infection [29]. Elevated antibacterial responses were also observed under prolonged cooling. For example, Ferguson and Sinclair [27] showed that overwintering *Eurosta solidagnis* larvae were characterized by an increased clearance of the gram-positive bacteria *Bacillus subtilis* in the hemolymph compared to autumn and spring larvae. According the present study, under cold conditions, insects may exhibit increased antibacterial responses during fungal infections.

We observed an increase in the expression of the gloverin and lysozyme genes in the midgut in response to fungal infection. This elevation was obviously caused by changes in the microbiota structure and the elevation in the bacterial load in the midgut during the development of mycosis. Similar changes were observed by Ramirez and coworkers [3] in the midgut of adult *Aedes aegypti* mosquitos in the acute stages of mycoses caused by *Beauveria* and *Isaria* species. However, we did not observe general temperature-dependent trends in the expression of antibacterial genes in the midgut.

The IAP gene was upregulated in the fat body at a low temperature and its upregulation in response to the infection was also observed only at a low temperature. Previous studies showed that IAP is linked to the IMD immune signaling pathway in insects [38]. In particular, knockdown of this gene in *D. melanogaster* led to confined expression of AMPs in response to bacterial infections and increased susceptibility to gram-negative bacteria [38]. In locusts, IAP knockdown led to blocked defensin expression, which was induced by *Metarhizium acridum* infection [37]. In our experiments, a lack of IAP expression at 25 °C was associated with a lower upregulation of antibacterial peptides and an active proliferation of bacteria in the hemolymph, which is consistent with the abovementioned studies.

The NOX-DUOX domain gene displayed an interesting pattern of expression. This system functions in the regulation of bacterial homeostasis, as has been shown in *Drosophila* and mosquitoes [40,42,80]. In our study, the gene was upregulated slightly in the fat body in response to fungal infection at both temperatures. In the midgut, we observed a significant downregulation of this gene at 15 °C (Table 2). This was correlated with a trend toward increased enterococci CFU counts in the midgut at 15 °C compared to 25 °C (Figure 3). This gene was downregulated in response to *C. militaris* infection only at 15 °C. This may be caused by the high acuity of mycosis at this temperature and it may be a consequence of the prioritization in immune reactions between the hemocoel and gut, as was suggested by Wei and coworkers [2]. However, this decrease in gene expression and the elevation in the enterococci load at 15 °C did not lead to the colonization of the hemocoel by bacteria, i.e., the proliferation of bacteria occurred only in the gut lumen under this temperature. Further immunological and histopathological studies are needed to establish the mechanisms of septicemia development during fungal infections.

We observed an upregulation of HSP70 in both tissues and an upregulation of HSP90 in the midgut at a low temperature. This result was expected because an increase in the expression of these genes during cold diapause has been observed in various insect taxa [46]. We also observed a downregulation in HSP70 expression in the fat body in response to *C. militaris* infection. Previous studies reported either an increase in HSP expression in different tissues of *G. mellonella* after infection with *B. bassiana* and *Conidiobolus coronatus*, or no change compared to uninfected insects [7,61,81]. These inconstancies may be caused by differences in pathogenesis that occur after infection with different fungal species and strains. Regarding the antibacterial response, it was shown that HSP70 transcripts were highly induced in arthropods (*Penaeus monodon*) after injection with bacteria *Vibrio* [82]. In wax moths, an increase in HSP90 expression was observed in response to *Bacillus thuringiensis* infection [83] and mixed (bacteria and yeast) infections [84]. Linder and coworkers [16] suggest that HSPs may improve immune functions against bacterioses at cool temperatures in *Drosophila melanogaster*. The authors have shown elevated expression of HSP83, PGRP-LS and AMPs, and increased resistance to bacteria (*Pseudomonas aeruginosa* and *Lactococcus lactis*) in cold conditions (17 °C) compared to warm conditions (29 °C). Similarly, in our work, septicemia was observed most often with the lowest levels of HSP expression (fungal infection at 25 °C), although we did not observe any correlations between HSP and AMP expression. It is possible that increased expression of HSPs at low temperature may help maintain tissue integrity in gut and other organs and prevent penetration of bacteria into hemolymph.

## 5. Conclusions

Bacterial associates of insects may influence the development and outcome of fungal infections. Using a model system of *C. militaris* and *G. mellonella*, in the present study we found that these interactions are significantly dependent on temperature. At high temperatures, these relationships develop in favor of spontaneous bacterioses, while under low temperatures they develop in favor of mycoses. The explanation for these outcomes may lie in the properties of the fungus, as well as in the immune reactions of the host during mycosis development. *C. militaris* is a weak competitor of bacteria and therefore it requires low temperatures to avoid antagonism with bacterial associates of the host to complete its development successfully. In addition, we observed weakened antifungal responses along with increased antibacterial responses in wax moths at a low temperature, which should be beneficial for the development of the fungus. We confirmed the previous works that have shown that AMP expression in *G. mellonella* is temperature-dependent [18,23,28]. However, a comparison of AMP expression in response to fungal infection at constant low (15 °C) and moderate (25 °C) temperatures was performed for the first time. Our results are consistent with previous studies in which short cooling of *G. mellonella* [28] and prolonged cooling of other lepidopterans [27] have led to an increase in antibacterial response. Moreover, we found increased expression of stress-related genes in the midgut under the constant low temperature, which may prevent the disruption of gut tissues and penetration of bacteria from the gut into the hemocoel. Further studies should focus on the interaction between bacterial growth and fungal infections using histopathological and histomolecular approaches, as well as on development of *C. militaris* in natural hosts using natural methods of infection. Our research may promote physiological and ecological studies into the interactions between pathogenic fungi, insect hosts and bacterial associates.

## Figures and Tables

**Table 1:** Two-way effects of *C. militaris* infection and temperature on the expression of AMP genes. Significant effects are highlighted in bold. Arrows show up- or downregulation of genes in response to infection and in response to cooling to 15 degrees. Arrows are shown only for significant (*p* < 0.05) and marginal (*p* = 0.05–0.10) effects.

	Effects		
	Infection	Temperature	Infection × Temperature
**Fat body**			
Galiomycin	**↑H_1,19_ = 14.3 *p* < 0.001**	H_1,19_ = 0.0 *p* = 1.00	H_1,19_ = 0.7 *p* = 0.40
Gallerimycin	**↑H_1,19_ = 14.3 *p* < 0.001**	H_1,19_ = 0.0 *p* = 0.88	H_1,19_ = 2.1 *p* = 0.15
Gloverin	**↑H_1,19_ = 14.3 *p* < 0.001**	H_1,19_ = 1.5 *p* = 0.23	H_1,19_ = 0.1 *p* = 0.82
Cecropin	↑H_1,19_ = 3.3 *p* = 0.08	**↑H_1,19_ = 3.9 *p* = 0.05**	H_1,19_ = 0.1 *p* = 0.76
Lysozyme	**↑H_1,23_ = 12.8 *p* < 0.001**	↑H_1,23_ = 3.2 *p* = 0.07	H_1,23_ = 0.1 *p* = 0.77
**Midgut**			
Galiomycin	**↑H_1,19_ = 10.1 *p* = 0.002**	H_1,19_ = 1.2 *p* = 0.29	H_1,19_ = 1.3 *p* = 0.27
Gallerimycin	**↑H_1,19_ = 14.3 *p* < 0.001**	H_1,19_ = 0.1 *p* = 0.76	H_1,19_ = 2.5 *p* = 0.11
Gloverin	**↑H_1,19_ = 6.2 *p* = 0.01**	H_1,19_ = 0.2 *p* = 0.65	H_1,19_ = 0.0 *p* = 0.88
Cecropin	H_1,23_ = 1.2 *p* = 0.27	H_1,23_ = 1.0 *p* = 0.33	H_1,23_ = 0.5 *p* = 0.49
Lysozyme	**↑H_1,23_ = 9.4 *p* = 0.002**	**↓H_1,23_ = 4.6 *p* = 0.03**	H_1,23_ = 1.1 *p* = 0.30

**Table 2:** Two-way effects of *C. militaris* infection and temperature on the expression of apoptosis, ROS and stress-related genes. Significant effects highlighted in bold. Arrows show up- or downregulation of genes in response to infection and in response to cooling to 15 degrees. Arrows are shown only for significant (*p* < 0.05) and marginal (*p* = 0.05–0.10) effects.

	Effects		
	Infection	Temperature	Infection × temperature
**Fat body**			
IAP	H_1,23_ = 0.8 *p* = 0.39	**↑H_1,23_ = 7.4 *p* = 0.007**	H_1,23_ = 3.0 *p* = 0.08
NOX-DUOX	**↑H_1,23_ = 4.3 *p* = 0.04**	H_1,23_ = 0.6 *p* = 0.45	H_1,23_ = 0.0 *p* = 0.86
Hsp70	**↓H_1,23_ = 4.6 *p* = 0.03**	**↑H_1,23_ = 14.5 *p* < 0.001**	H_1,23_ = 0.0 *p* = 0.95
Hsp90	↑H_1,23_ = 2.8 *p* = 0.09	H_1,23_ = 0.3 *p* = 0.56	H_1,23_ = 0.1 *p* = 0.77
**Midgut**			
IAP	**↑H_1,23_ = 7.7 *p* = 0.006**	H_1,23_ = 0.0 *p* = 0.91	H_1,23_ = 0.0 *p* = 0.91
NOX-DUOX	H_1,23_ = 1.8 *p* = 0.18	**↓H_1,23_ = 6.2 *p* = 0.01**	H_1,23_ = 1.3 *p* = 0.25
Hsp70	H_1,23_ = 0.0 *p* = 0.93	**↑H_1,23_ = 13.7 *p* < 0.001**	H_1,23_ = 0.1 *p* = 0.71
Hsp90	H_1,23_ = 0.2 *p* = 0.64	**↑H_1,23_ = 8.7 *p* = 0.003**	H_1,23_ = 0.8 *p* = 0.39

## Supplementary Materials

The following are available online at https://www.mdpi.com/2309-608X/6/3/170/s1, **Figure S1.** Rarefaction curves of the OTU numbers for each sample. **Table S1.** List and description of genes and primer sequences used in qPCR. **Figure S2.** Diversity indexes of bacterial communities in the midgut of wax moth larvae at 96 h post injection of *С. militaris* (2500 conidia per larva) with incubation at 15 °C and 25 °C. Indexes were calculated for OTU levels. Different letters indicate significant differences between treatments (Dunn’s test, *p* < 0.05). **Figure S3.** Wax moth larvae overgrown with *B. bassiana* (A) and *M. robertsii* (B) at four days after injection with 2500 conidia per larva and incubation at 25 °C.
