# Supplementary material for: Interplay between Fungal Infection and Bacterial Associates in the Wax Moth Galleria mellonella under Different Temperature Conditions
Source: J Fungi (Basel). 2020 Sep 10;6(3):170. doi: 10.3390/jof6030170 (PMC7558722; doi:10.3390/jof6030170)
Supplement: Supplementary file 1 [file jof-06-00170-s001.zip › Appendix A.pdf]

ELECTRONIC SUPPLEMENTARY MATERIAL

Interplay between fungal infection and bacterial associates in the wax moth *Galleria mellonella* under different temperature conditions

Vadim Yu Kryukov[1], Elena Kosman[1], Oksana Tomilova[1], Olga Polenogova[1], Ulyana Rotskaya[1], Maksim Tyurin[1], Tatyana Alikina[2], Olga Yaroslavtseva[1], Marsel Kabilov[2], Viktor Glupov[1]

[1] Institute of Systematics and Ecology of Animals SB RAS, Frunze str. 11, 630091 Novosibirsk, Russia; krukoff@mail.ru

[2] Institute of Chemical Biology and Fundamental Medicine SB RAS, Lavrentiev av. 8, Novosibirsk 630090, Russia; kabilov@niboch.nsc.ru

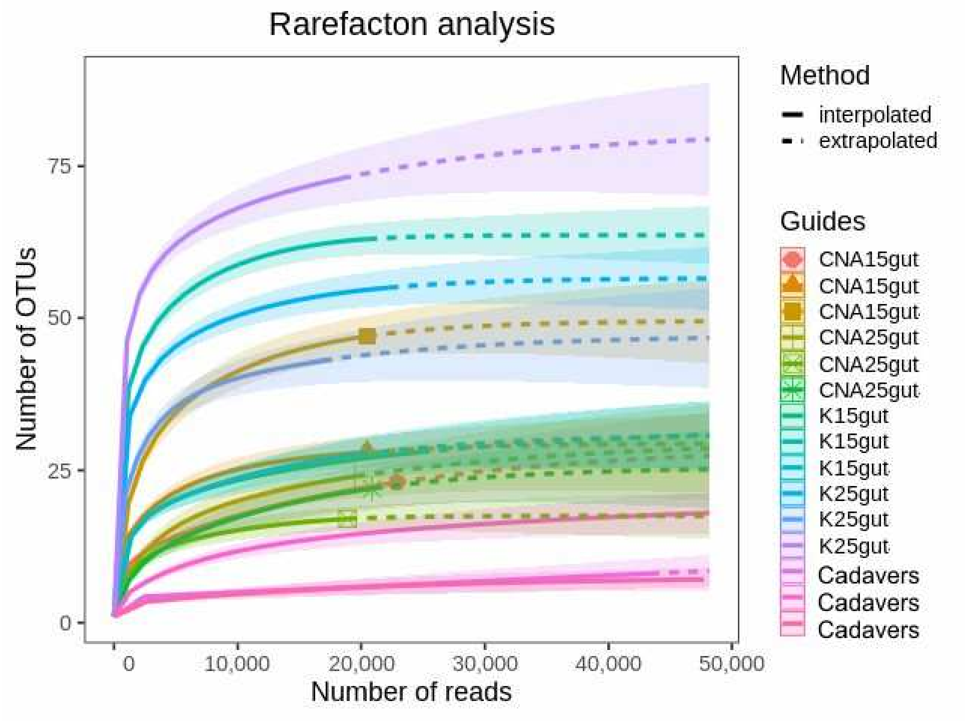

Figure S1. Rarefaction curves of the OTU number for each sample.
CNA - infection with *C. militaris*, K - control, 15 and 25 - temperature °C, cadavers - bacterial decomposed larvae.

Table S1. List and description of genes and primers sequences used in the qPCR.

Gene Name	NCBI genebank Accession number	Gene symbol	Primer sequence (5' – 3')	Product size (bp)	PCR efficiency (±SD)	Tm in qPCR, ^{o}C	Primers source
Translation elongation factor 1-alfa	AF423811.1	EF1a	For CTGAACCTCCTTACAGTGAATCC<br>Rev GCATGTTATCTCCGTGCCAG	135	1,96±0,1/<br>1,93±0,06/<br>1,90±0,04	60/62/64	Melo et al., (2013) [b]<br>*modified
DNA-directed RNA polymerase II subunit RPB11	XM_026902197.2	RBP11	For CGCCAACCTTTGAATCATTCCTT<br>Rev TGGTGTCTGATCATGTTTCCAAGA	136	2,04±0,01/<br>2,06±0,02/<br>1,97±0,1	60/62/64	Rotskaya U.N. [c]
NOX-DUOX domain	XM_026901961.2	NOX-DUOX	For AGAGTGGCATCCCTTTACTATTGTG<br>Rev CCGTCCAATCACCTTTGACTCG	92	1,95±0,06	64	Lange et al., (2018) [a]
Gallerimycin	AF453824.1	Gal	For GAAGTCTACAGAATCACACGA<br>Rev ATCGAAGACATTGACATCCA	161	1,7±0,001	62	Melo et al., (2013) [b]
Gloverin	AF394588.1	Glo	For AGATGCACGGTCCTACAG<br>Rev GATCGTAGGTGCCTTGTG	93	1,91±0,02	62	Melo et al., (2013) [b]
Galiomycin	AY528421.1	Glm	For GTGCGACGAATTACACCTC<br>Rev TACTCGCACCAACAATTGAC	103	2,07±0,02	62	Melo et al., (2013) [b]
Inhibitor of apoptosis protein	FJ643490.1	IAP	For ACTTTCAACGATTGGCCGCT<br>Rev TCCCAATCTTTAAGACCGCCG	128	1,94±0,02	64	Rotskaya U.N. [c]
Heat shock protein 70	XM_031909614.1	Hsp70	For CGACGACCCCAAGATACAACAG<br>Rev CGTCTCGCCCTTGAACTCC	100	1,95±0,01	64	Rotskaya U.N. [c]
Heat shock protein 90	AF394591.1	Hsp90	For TCAGCTTCACGGACAGCTTCT<br>Rev GACCCCAGAGCTTGCATTGG	152	2,02±0,01	62	Rotskaya U.N. [c]
Cecropin-like	XM_026898304.2	Cec	For CTGTTCGTGTTCGCTTGTGT<br>Rev GTGGTAGGCGAAGCAGCTAC	158	1,99±0,02	64	Lange et al., (2018) [a]
Lysozym-like	XM_026894466.2	Lys	For GGACTGGTCCGAGCACTTAG<br>Rev CACGGTTGCCTCTAAATGCG	253	1,93±0,02	60	Lange et al., (2018) [a]

*modified – primers were modified by Rotskaya U.N.

[a] Lange et al, Frontiers in Immunology (2018) https://doi.org/10.3389/fimmu.2018.02114

[b] Melo et al., *PlosOne*, Dataset, (2013). 10.1371/journal.pone.0078905

[c] Primers were designed in the Laboratory of Insect Pathology ISEA SB RAS with an online resource, https://www.ncbi.nlm.nih.gov/tools/primer-blast/, and their properties were estimated by IDT OligoAnalyzer 3.1 (http://eu.idtdna.com/calc/analyzer).

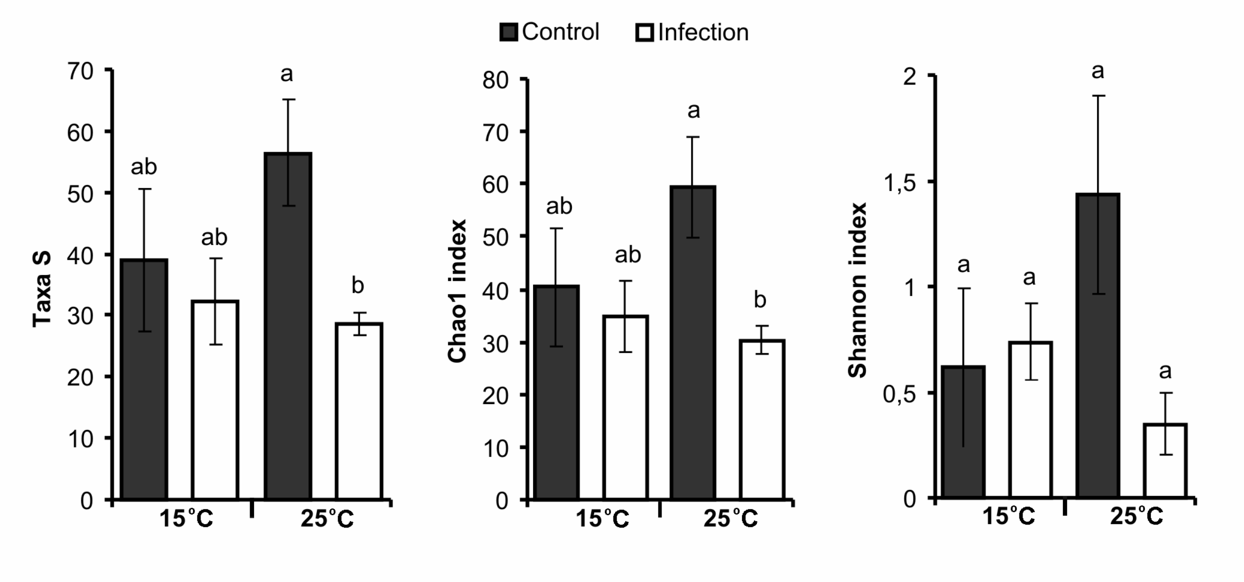

Figure S2. Diversity indexes of bacterial communities in midgut of wax moth larvae at 96 h post injection of *C. militaris* (2500 conidia per larva) and the incubation under 15ºC and 25ºC .Indexes were calculated for OTU level. Different letters indicate significant differences between treatments (Dunn's test, $P < 0.05$).

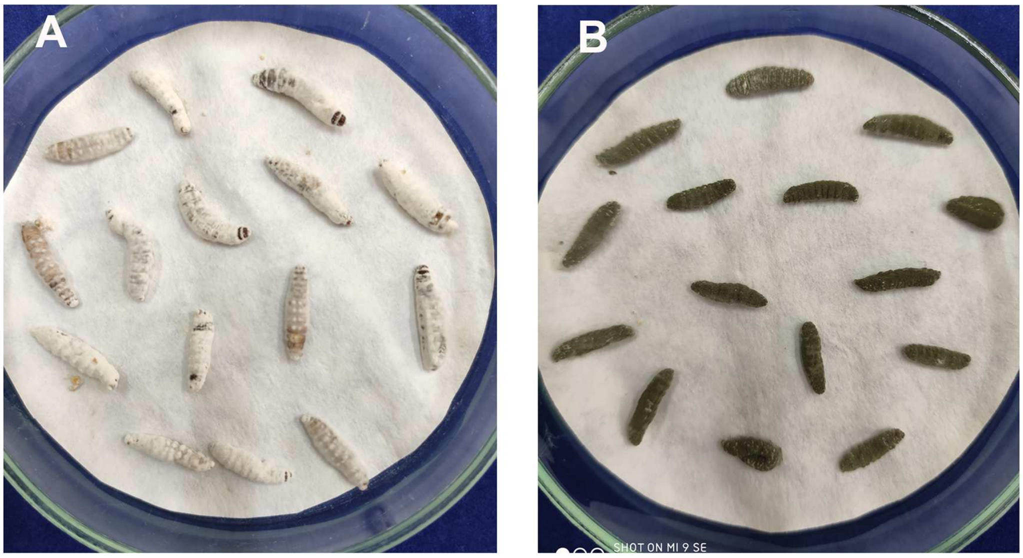

Figure S3. Wax moth larvae overgrowing with *B. bassiana* (A) and *M. robertsii* (B) at four day after injection with 2,500 conidia per larva and incubation under 25ºC.
